# Nanogels of Succinylated Glycol Chitosan-Succinyl Prednisolone Conjugate: Preparation, In Vitro Characteristics and Therapeutic Potential

**DOI:** 10.3390/pharmaceutics11070333

**Published:** 2019-07-13

**Authors:** Haiyan Zhou, Atsuko Ichikawa, Yuri Ikeuchi-Takahashi, Yoshiyuki Hattori, Hiraku Onishi

**Affiliations:** 1 Department of Drug Delivery Research, Hoshi University, 2-4-41, Ebara, Shinagawa-ku, Tokyo 142-8501, Japan; 2 CMIC Pharma Science Co., Ltd., 10221, Kobuchisawacho, Hokuto 408-0044, Yamanashi, Japan

**Keywords:** succinylated glycol chitosan-succinyl prednisolone conjugate, nanogel, viability, cellular uptake, in vivo efficacy, toxic side effect

## Abstract

A novel anionic nanogel system was prepared using succinylated glycol chitosan-succinyl prednisolone conjugate (S-GCh-SP). The nanogel, named NG(S), was evaluated in vitro and in vivo. S-GCh-SP formed a nanogel via the aggregation of hydrophobic prednisolone (PD) moieties and the introduced succinyl groups contributed to the negative surface charge of the nanogel. The resultant NG(S) had a PD content of 13.7% (*w*/*w*), was ca. 400 nm in size and had a ζ-potential of −28 mV. NG(S) released PD very slowly at gastric pH and faster but gradually at small intestinal pH. Although NG(S) was easily taken up by the macrophage-like cell line Raw 264.7, it did not decrease cell viability, suggesting that the toxicity of the nanogel was very low. The in vivo evaluation was performed using rats with trinitrobenzene sulfonic acid (TNBS)-induced colitis. NG(S) and PD alone were not very effective at 5 mg PD eq./kg. However, NG(S) at 10 mg PD eq./kg markedly suppressed colonic damage, whereas PD alone did not. Furthermore, thymus atrophy was less with NG(S) than with PD alone. These results demonstrated that NG(S) is very safe, promotes drug effectiveness and has low toxicity. NG(S) has potential as a drug delivery system for the treatment of ulcerative colitis.

## 1. Introduction

Ulcerative colitis (UC), a severe inflammatory bowel disease (IBD), is a chronic relapsing disease that causes colonic inflammation and various complications [1,2,3]. Although its etiology remains unclear, UC is regarded as an autoimmune disease. Previous studies reported that UC is induced by genetic, inherited, environmental and behavioral factors [4,5]. UC is also associated with imbalances in the enteric flora and intestinal immune system.

Pharmacotherapy plays an important role in the treatment of UC, the therapeutic goal of which is the alleviation of inflammation and mucosal healing. Anti-inflammatory and immunosuppressive agents are the standard treatment for UC [6,7,8]. Amino salicylic acid-related medicines, anti-inflammatory glucocorticoids, immunosuppressive drugs and biologics against pro-inflammatory cytokines are generally administered according to the disease state and severity [9,10]. Glucocorticoids are often selected because they rapidly exert strong anti-inflammatory effects and are effective in the treatment of severe disease states. However, steroidal drugs may cause serious adverse effects, such as diabetes, osteoporosis and glaucoma [11,12,13].

Drug delivery systems (DDSs) have recently been investigated for their ability to enhance therapeutic efficacy and reduce adverse effects. In UC therapy involving steroidal drugs, several DDSs have been used to promote drug efficacy and reduce toxic side effects [14,15,16,17,18,19]. These DDSs have been developed to efficiently deliver drugs to diseased areas and suppress systemic absorption. Systemic absorption is directly related to many toxic side effects. Therefore, it is very important to deliver the active agents specifically to the diseased area. In addition, DDSs need to give the efficient concentrations for the reasonable periods.

Nanoparticulate dosage forms (NPs) have attracted attention as DDSs for the treatment of UC. NPs penetrate the mucus layer in the intestinal region, accumulate at the sites of colitis and are not subjected to rapid elimination by diarrhea associated with UC [19,20]. Furthermore, NPs have been reported to accumulate well at sites of UC due to the epithelial enhanced permeability and retention (eEPR) effect, which is attributed to mucosal dysfunction, high epithelial permeability and the marked infiltration of immune cells. Therefore, NPs with the ability to prolong drug release are very promising for specific delivery and increased drug exposure at UC sites [14,18]. 

A novel NP system loaded with prednisolone (PD), the most frequently used glucocorticoid in the treatment of UC [21,22], was developed in the present study. We recently demonstrated that water-soluble macromolecule-hydrophobic drug conjugates may easily form as nanogels under high drug-loading conditions [23]. Furthermore, these conjugates with a labile combination, such as carboxyl esters, generally ensure prolonged release due to their own sensitivities to a number of factors, including pH [24,25,26]. Glycol chitosan (GC)-succinyl prednisolone (GP) conjugate, named GCh-SP, with high drug loading was synthesized based on these findings. 

Recent pathophysiological studies revealed that positively charged proteins accumulate in the inflamed colonic mucosa, while negatively charged particles or gels preferentially concentrate in diseased colonic membranes [27]. In addition, since negativelycharged NPs are not subjected to mucoadhesion in the upper gastrointestinal tract, stomach or upper small intestinal mucosa, they are useful for targeting inflamed regions in UC [28]. Therefore, GCh-SP has been modified into a negativelycharged conjugate, namely, the succinylation of GCh amino groups was performed. The conjugate obtained, succinylated GCh-SP, named S-GCh-SP, was prepared as a target DDS conjugate. S-GCh-SP with high drug loading forms a nanogel due to hydrophobic PD moieties and functions as a negativelycharged NP because of the presence of succinyl groups in the water-soluble polymer parts. 

The preparative procedure of S-GCh-SP NP, its particle characteristics and in vitro release characteristics were described in the present study. The biological features of toxicity and cellular interactions were examined in vitro and efficacy was investigated in vivo using animal models of UC.

## 2. Materials and Methods 

### 2.1. Materials

6-O-glycol chitosan (GCh; MW = ca. 100,000, deacetylation degree = approximately 80% (mol/mol)), PD and succinyl prednisolone (SP), succinic anhydride (SA), 1-(3-dimethylaminopropyl)-3-ethylcarbodiimide hydrochloride (WSC), fluorescein isothiocyanate (FITC) and fluorescein (FTC) were purchased from Wako Pure Chemical Industries, Ltd. (Osaka, Japan). Lipopolysaccharide (LSP) and trinitrobenzene sulfonic acid (TNBS) were purchased from Sigma Chemical Company (St. Louis, MO, USA). Sephadex G50 (fine grade) was obtained from GE Healthcare Bio-Sciences AB (Uppsala, Sweden). All other chemicals were of reagent grade. 

### 2.2. Instruments

UV-VIS absorption was performed using a Beckman DU640 spectrophotometer (Beckman Coulter, Inc., Brea, CA, USA). NMR spectra were measured with a JNM-ECA600 II spectrometer (JEOL, Tokyo, Japan). A Shimadzu SALD-7100 nanoparticle size analyzer was used to assess particle sizes. The γ-potentials of particles were measured using an ELS-Z2 apparatus (Otsuka Electronic Co., Ltd., Osaka, Japan). 

### 2.3. Preparation of GCh-SP and S-GCh-SP

**Figure 1:**
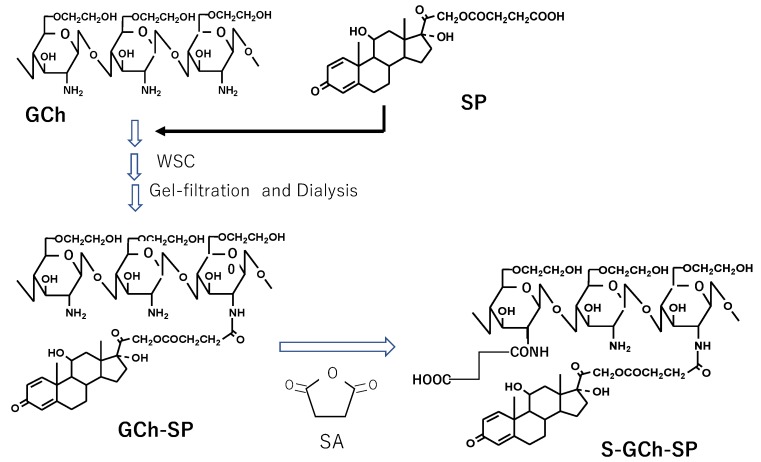
Synthetic scheme of GCh-SP and S-GCh-SP. GCh: glycol-chitosan, SP: succinyl-prednisolone, GCh-SP: GCh-SP conjugate, S-GCh-SP: succinylated GCh-SP, WSC: water-soluble carbodiimide, SA: succinic anhydride.

The preparative scheme of GCh-SP and S-GCh-SP is shown in Figure 1. GCh and SP were coupled using WSC; GCh (100 mg) was dissolved in 15 mL water and 5 mL tetrahydrofuran (THF) solution containing SP (100 mg) was added. After the pH value of the resulting mixture was adjusted to 6.2–6.3 using 1 M HCl and 1 M NaOH, WSC (500 mg) was added under ice cooling conditions and then stirred for 1 h. The reaction continued for another 23 h. The resultant solution was then chromatographed using a Sephadex G50 column (1.6 cm inner diameter × 25 cm length) with 0.1 M NaCl as the elution solvent. Macromolecular fractions were collected and dialyzed extensively against water using a cellulose tube. The residue in the tube was obtained as an aqueous suspension of the nanogel of GCh-SP, named NG(G). In the second step, NG(G) was modified chemically with SA, in which the amino groups of the GCh parts underwent succinylation based on the findings reported by Hamada et al. [29] and Kato et al. [30]. THF as well as 0.5 M phosphate buffer (pH 7) was added to aqueous medium containing NG(G) (150 mg) at a ratio of 1/3 (*v*/*v*) and an excess amount of SA (1050 mg) was added gradually and stirred for 3 h. Throughout the reaction period, the pH of the reaction mixture was adjusted to 7 using 1 M HCl and 1 M NaOH and temperature was maintained at 15 °C. The reaction mixture was dialyzed using a cellulose tube against excess water and the remaining mixture in the tube was chromatographed using a Sephadex G50 column with 0.1 M NaCl as the elution solvent. High-molecular-weight fractions were collected and dialyzed in a similar manner. The resultant mixture obtained was an aqueous suspension of a nanogel of S-GCh-GP, named NG(S). 

The chemical structures of GCh and S-GCh-SP were examined based on ^1^H-NMR spectra using a mixture of DMSO-d_6_/D_2_O (1:1, *v*/*v*) as a solvent, in which tetramethylsilane (TMS) was added as a standard (0 ppm) of the proton signal.

The PD contents of GCh-SP and S-GCh-SP were examined as follows: GCh-SP and S-GCh-SP were added to 0.1 M NaOH aqueous solution. After the mixture was heated at 45 °C for 10 min, it was centrifuged, and the supernatant was measured spectrophotometrically at 246 nm. The content of PD was calculated from the absorbance of free PD in 0.1 M NaOH aqueous solution.

### 2.4. Preparation of FITC-Labeled NG(S)

NG(S) was modified with FITC. FITC (0.5 mg), dissolved in 0.5 mL dimethylsulfoxide (DMSO), was added to a suspension of NG(S) (10 mg) in 5 mL of 0.5 M phosphate buffer at pH 7. The mixture was stirred at 4 °C overnight in the dark. The mixture was subjected to gel filtration using a Sephadex G50 column (1.6 cm inner diameter × 25 cm length) with phosphate-buffered saline at pH 7.4 (PBS) as the elution solvent and macromolecular parts were collected and dialyzed against water. The mixture obtained was used as a suspension of FITC-labeled NG(S), named FTC-NG(S).

### 2.5. Particle Characteristics

Particle sizes were measured and the ζ-potentials of nanogels were investigated by dynamic light scattering using the ELS-Z2 apparatus. 

### 2.6. In Vitro Release Studies

The aqueous suspensions (2 mL) containing NG(S) at 0.43 mg/mL in Japanese Pharmacopoeia (JP)-17 first fluid (pH 1.2) or its second fluid (pH 6.8) were prepared by simple mixing of media. The suspensions were incubated at 37 °C by horizontal shaking at 60 rpm. The aliquot samples (50 μL) were taken at appropriate time points. Immediately after each sampling, 100 μL of 0.1 M acetate buffer (pH 4) was mixed to each sample taken, to suppress the further release. The resultant mixture was analyzed by HPLC to determine the released PD. 

### 2.7. HPLC Assay

HPLC was performed at room temperature. Regarding the apparatus, a Shimadzu LC-6AD pump was used with a Shimadzu SPD-10AV VP UV-VIS detector set at a wavelength of 246 nm and Shimadzu C-R7A plus Chromatopac (Kyoto, Japan). A YMC Pack ODS-AM column (I.D. of 6 mm, length of 150 mm; YMC Co., Ltd., Kyoto, Japan) was used as the analytical column. A mixture of acetonitrile and 50 mM citrate buffer with pH adjusted to 4.1 with phosphoric acid (35:65, *v*/*v*) was used as the mobile phase. The flow rate was set at 1 mL/min with an injection volume of 20 μL. PD concentrations were assessed using an absolute calibration curve.

### 2.8. Cell Culture

Raw 264.7 cells were donated by Prof. Kei-ichi Ozaki (Education and Research Center for Pharmaceutical Sciences, Osaka University of Pharmaceutical Sciences, Osaka, Japan) and used as cells in in vitro studies. Cells were cultured in Dulbecco’s modified Eagle’s medium (DMEM) with 10% heat-inactivated fetal bovine serum (FBS) and kanamycin (100 μg/mL) in a humidified atmosphere containing 5% CO_2_ at 37 °C.

### 2.9. In Vitro Viability Studies Using Raw 264.7 Cells in the Presence of Several Substances

Raw 264.7 cells were seeded separately at a density of 1 × 10^4^ cells per well in 96-well plates and maintained in DMEM supplemented with 10% FBS for 24 h before the treatment. Cells were treated with the media of PD alone, NG(S) and FTC-NG(S), at the concentration of PD, equivalent to 5, 20 and 80 μg/mL and then incubated for another 24 h. As a control for NG(S), succinylated GCh (S-GCh) was used at the same weight concentration of NG(S); S-GCh was produced by treating NG(S) with 0.1 M NaOH to completely remove PD. Cell numbers were counted with Cell Counting Kit-8 (Dojindo Laboratories, Kumamoto, Japan). Cell viability was expressed relative to the absorbance at 450 nm of untreated cells. 

### 2.10. Cellular Uptake of FTC-NG(S)

Raw 264.7 cells were cultured in a 35-mm dish (5 × 10^5^ cells per well) with or without 1 μg/mL LPS for 18 h. FTC-NG(S) or FTC alone was added to the cultures at a concentration of 1 μg FTC eq./mL and this was followed by an incubation for 1 h. Cells were then washed with PBS three times and fixed with 4% formaldehyde in PBS (Mildform 10N, Wako, Osaka, Japan) at room temperature for 15 min. Cells were observed with a confocal laser scanning microscope (FV1200, Olympus Corp. Tokyo, Japan) at excitation and emission wavelengths of 495 and 520 nm, respectively.

### 2.11. Animals

Male Wistar rats (7 weeks old, 200–210 g) were purchased from Tokyo Laboratory Animal Science Co., Ltd. Animals were kept on the breeding diet MF supplied by Oriental Yeast Co., Ltd. (Tokyo, Japan) with water available ad libitum in a room in which temperature and relative humidity were maintained at 23 ± 1 °C and 60 ± 1%, respectively. The light-dark cycle was 12 h. The experimental protocol was approved by the committee on Animal Research of Hoshi University (Tokyo, Japan; project identification code: 26-038, approval date: April 20, 2018) and animal experiments were performed according to the Guiding Principles for the Care and Use of Laboratory Animals of Hoshi University.

### 2.12. In Vivo Studies on Efficacy and Toxic Side Effects

**Figure 2:**
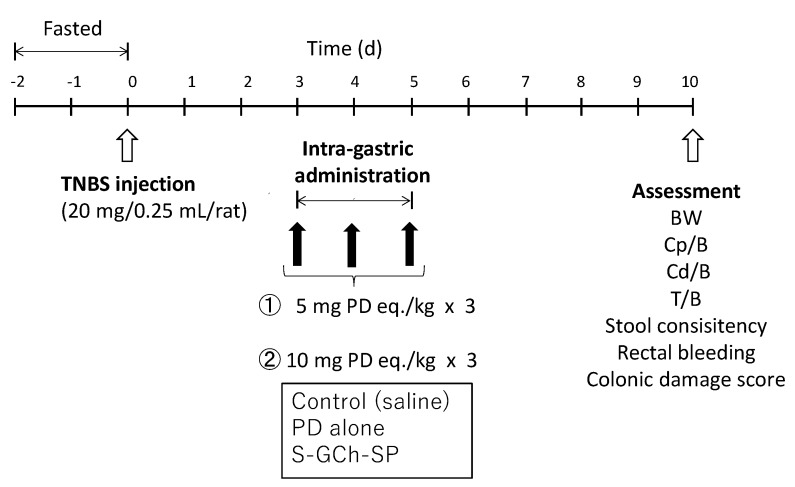
Animal experiment procedure to test the efficacy and toxic side effect. BW: body weight, B: body weight, Cp: proximal colon weight, Cd: distal colon weight, T: thymus weight.

UC animal models were produced by the treatment of rats with TNBS [14,31]. Animal experimental schedules were performed as shown in Figure 2. TNBS was dissolved in 50% (*v*/*v*) ethanol aqueous solution at a concentration of 80 mg/mL. After rats were fasted for 2 days, the TNBS solution (0.25 mL) was instilled into the colon of each rat, 8 cm from the anus using a catheter 26–28). Three days after the TNBS treatment, rats were divided into the saline (control), PD alone and NG(S) groups. Drugs were administered at 5 and 10 mg PD eq./kg via gastric intubation once daily for three consecutive days. Five days after the final administration, animals were sacrificed by excessive ether anesthesia and the colon and thymus were excised. 

Therapeutic efficacy was examined from the ratio of (proximal colon weight: Cp)/(body weight: B), Cp/B and (distal colon weight: Cd)/(body weight: B), Cd/B. The severity of inflammation was examined as follows: Stool consistency (SC), rectal bleeding (RB) and colonic damage were observed visually [13]. SC was scored as 0, 2 and 4 for the pellet form, soft form (not stuck to the anus) and liquid form (stuck to the anus), respectively. The scores for RB were set as 0, 2 and 4 for the states of no blood, slight bleeding and gross bleeding. Regarding the damage to the diseased site, colonic damage scores (CDS) were assessed as follows: 0, no damage; 1, hyperemia with no ulcers; 2, one small ulcer with no inflammation; 3, one small ulcer with inflammation; 4, two or more small ulcers with inflammation; 5, ulcers including a large (>1 cm) ulcer with inflammation [32]. The ratio of (thymus weight: T)/(body weight: B) was investigated as an index of the toxic side effects of PD [15,17].

### 2.13. Histopathological Study

Colon tissue samples were collected from the control, PD alone and NG(S) groups, administered 10 mg PD eq./kg × 3 in efficacy studies. Colonic parts, including ulcers and/or inflammation, were collected and cut open. They were fixed in 10% buffered formalin and embedded in paraffin. Cross-sections at a thickness of 5 μm were cut using a microtome. The samples obtained were stained with hematoxylin and eosin and observed under a light microscope. Low-magnification images of typical examples were photographed.

### 2.14. Statistical Analysis

Data were compared using ANOVA followed by Dunnett’s post hoc test. In all cases, a significant difference was set as *p* < 0.05. 

## 3. Results and Discussion

### 3.1. Preparation and Characterization of GCh-SP and S-GCh-SP

GCh-SP was prepared using a previously described technique with slight modifications. In the present study, SP was used in the non-salt form and the ratio of SP/GCh was 1, higher than the previous ratio of 0.5. The reaction conditions and PD content of the obtained GCh-SP are shown in Table 1. The PD content of GCh-SP was more than 20% (*w*/*w*). GCh-SP displayed white turbidity in water and was translucent with the addition of organic solvents, such as THF and methanol. This was attributed to hydrophobic PD moieties, while only slight turbidity was observed in the previous study and most conjugates were obtained in the soluble form. 

S-GCh-SP was prepared by the succinylation of the amino groups of GCh-SP. The reaction conditions and PD content of the obtained S-GCh-SP are shown in Table 2. The drug content of PD was less than that of GCh-SP. This decrease in the PD content was based on the derivatization of the succinyl groups and the smaller liberation of conjugated PD in the preparation process. 

**Figure 3:**
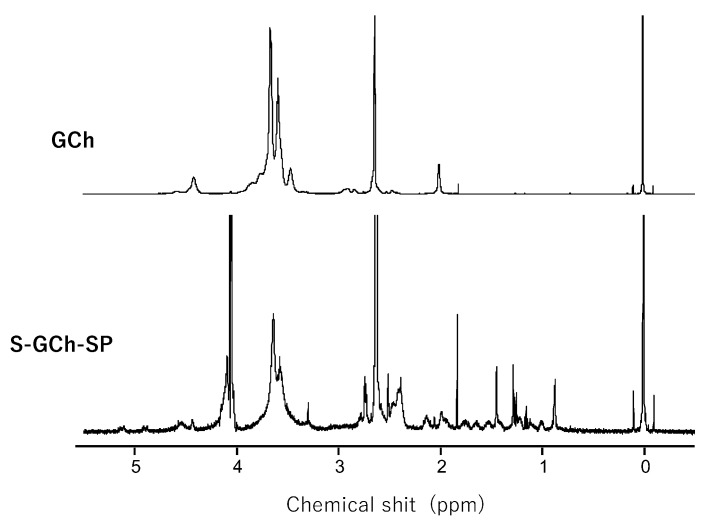
^1^H-NMR spectra of GCh-SP and S-GCh-SP. The mixture of dimethylsulfoxide (DMSO)-d_6_/D_2_O (1:1, *v*/*v*) was used as a solvent.

The ^1^H-NMR charts of GCh and S-GC-SP are shown in Figure 3. The protons of the acetyl group, glycol protons and the H-1 protons of sugar units were detected in the regions of approximately 2.0, 3.4–3.8 and 4.3–4.6 ppm, respectively. Based on the integrated intensities of these protons in the ^1^H-NMR spectra of GCh, the deacetylation degree and glycol substitution one was calculated to be 24 and 100% (mol/mol) per sugar unit, respectively. Furthermore, regarding the spectrum of S-GCh-SP, the proton signals belonging to the PD moieties were widely observed. The drug content calculated from the proton signals derived from PD were consistent with the values obtained from UV absorption. Furthermore, the signals of succinyl protons were in the range of 2.2–2.5 ppm. Based on their integrated intensities, total succinyl group contents were 64% (mol/mol) per sugar unit and were composed of free and PD-combined succinyl groups.

### 3.2. Particle Characteristics of NG(G) and NG(S)

**Figure 4:**
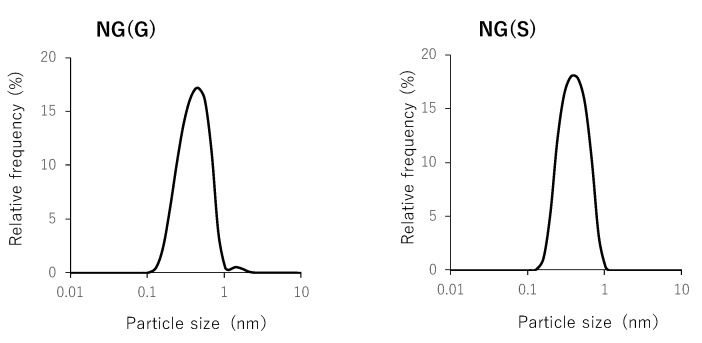
Size distribution of nanogels.

GCh-SP and S-GCh-SP gave white turbid suspensions in water. Based on the analogy of the conjugate between many reactive groups with water-soluble polymers and hydrophobic small molecules, they were nanogels called NG(G) and NG(S), respectively. Their size distributions were obtained as described in Figure 4. Particle characteristics were summarized in Table 3. NG(G) showed a size of nearly 400 nm, ranging between 150 and 1000 nm. The ζ-potential was a relatively high positive value of more than 40 mV, which was due to the remaining amino groups of GCh. NG(S) had a similar particle size to that of NG(G), although the PD content was smaller in NG(S). The compact nanogel form was maintained at a PD content of 13.7% (*w*/*w*). The ζ-potential was fairly negative, which was mainly attributed to the derivatization of succinyl groups to the amino groups of GCh.

### 3.3. Characteristics of FTC-NG(S) 

FTC-NG(S) was obtained by the introduction of FTC to the amino groups of GCh parts. The reaction was conducted at the FITC/NG(S) ratio of 0.1 (*w*/*w*). The content of FTC in FTC-NG(S) was 0.9% (*w*/*w*), which was based on its absorbance at 495 nm with the absorbance of FTC as a standard.

### 3.4. In Vitro Release from NG(S) 

First, the validity of the present method was checked as follows: Namely, the samples taken at 1 h and 7 h after the start of the incubation were treated using an ultrafilter with cut-off MW of 100,000 (Microcon, made by Millipore Corp., Bedford, MA, USA) and the filtrate was analyzed by HPLC. The resultant PD concentration was the same as the concentration given by the present method. Therefore, the present method was considered to give the concentration of free PD released out of NGs, indicating it was valid. 

**Figure 5:**
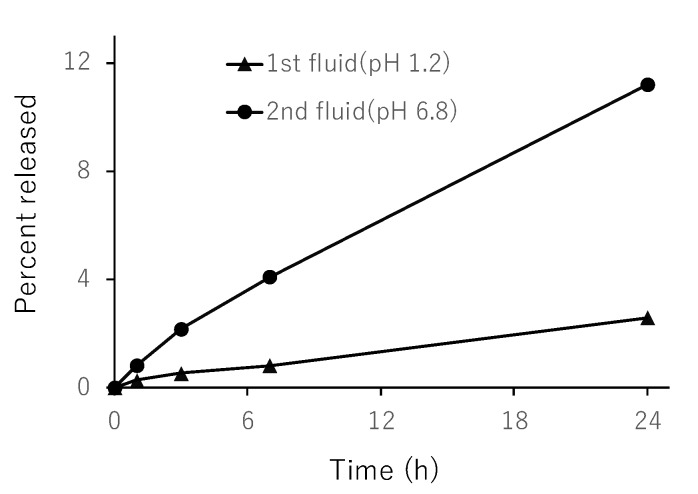
Release of prednisolone (PD) from nanogel (NG(S)) in the JP-17 1st and 2nd fluids at 37 °C. The results are expressed as the mean ± S.D. (*n* = 3).

The release profiles were obtained as shown in Figure 5. As the hydrophilic macromolecules in NG(S) were swollen by the immersion of aqueous media, the PD liberated by the ester hydrolysis was released into the media. As to the ester hydrolysis, the following experiment was performed. After methanol was added to the taken sample at the ratio of 1:1 (*v*/*v*), the obtained mixture was analyzed by HPLC. The resultant PD concentration was almost the same as the concentration of the released PD given by the present release study. This suggested that, even though the PD solubility was raised much higher by the addition of methanol, the resultant concentration of free PD was almost the same as the concentration of the released PD in the release studies. It was presumed that the PD released out of NG might be almost equivalent to the free PD caused by the ester hydrolysis. Release was very slow at pH 1.2, at which the release ratio was less than 4% (*w*/*w*) even at 24 h. At pH 6.8, release was accelerated and the release ratio was nearly 12% (*w*/*w*) after 24 h. These results suggested that the liberation of PD from NG(S) was suppressed in the stomach. However, PD was released gradually under intestinal pH conditions at a greater speed than that in the stomach. The release patterns were considered appropriate for the delivery to the lower intestine.

### 3.5. In Vitro Viabilities of Raw 264.7 Cells in Media with Different Substances

**Figure 6:**
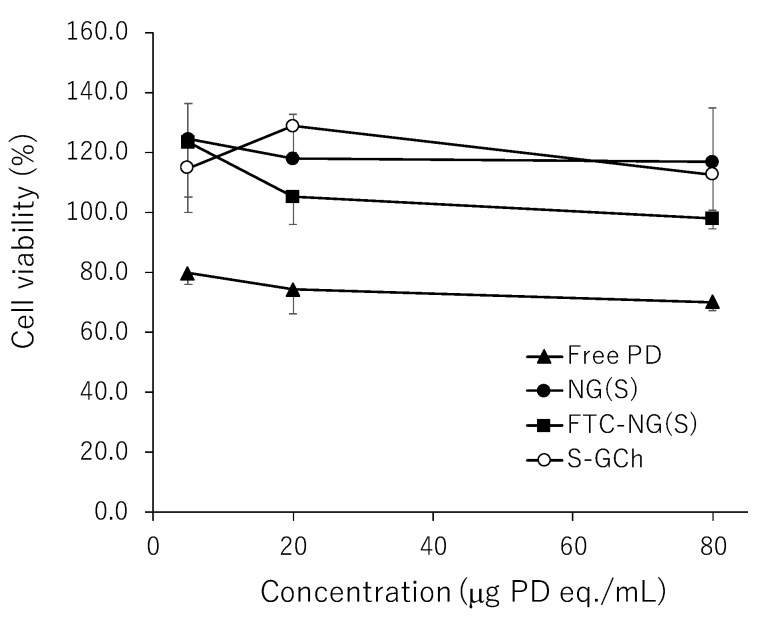
Viability of Raw 264.7 cells after the exposure to different substances for 24 h. Viability was calculated relative to that of untreated cells (100%). The results are expressed as the mean ± S.D. (*n* = 3–4).

As macrophages are importantly related to biological reactions in the inflammatory regions, macrophage-like cells, Raw 264.7 cells, were used in the studies of cytotoxicity and cellular uptake. The cytotoxicity of NG(S) was examined using cultured Raw 264.7 cells. The effects of PD alone, NG(S), FTC-NG(S) and the nanogel carrier (S-GCh) on cell viability were investigated in the range of 5–80 µg PD eq./mL. The results obtained are shown in Figure 6. The toxicity of PD alone was weak. S-GCh exhibited no toxicity. Furthermore, the exposure to NG(S) and FTC-NG(S) only negligibly decreased cell viability. S-GCh and NG(S) increased cell proliferation to some extent (100–120%). These results suggested that NG(S) and FTC-NG(S) are non-toxic substances for cultured Raw 264.7 cells and appear to be very safe.

### 3.6. Cellular Uptake of FTC-NG(S) by Raw 264.7 Cells

**Figure 7:**
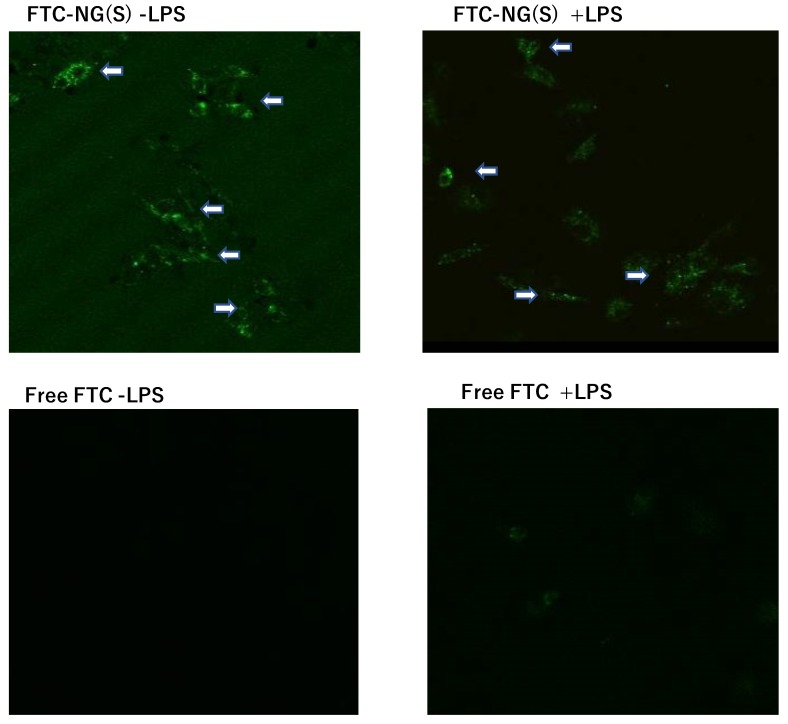
CLSM images of Raw 264.7 cells after incubation with FTC-NG(S) and FTC. The substances were added at the concentration of 1 μg FTC eq./mL. All the photos are shown in the same scale. − LPS: no LPS, + LPS: LPS added at 1 μg/mL. The white arrows show the uptake of FTC-NG(S) by the cells.

In order to investigate the uptake profiles of NG(S) by Raw 264.7 cells, the fluorescence probe FTC was introduced into the nanogel. FTC-NG(S) clearly displayed a fluorescence-emitting spot at an excitation wavelength of 490 nm and emission wavelength of 520 nm. In uptake tests, FTC alone was used as a control with the same concentration of FTC moieties of FTC-NG(S). The results obtained are shown in Figure 7. Images showed that FTC-NG(S) nanogels were distributed in the cytoplasm. On the other hand, fluorescence was hardly detected in FTC alone. The addition of LPS did not markedly affect the cellular uptake of FTC-NG(S). FTC-NG(S) appeared to be easily taken up irrespective of cellular activation. 

### 3.7. Efficacy and Toxic Side Effects of NG(S) in Rats with TNBS-Induced UC

**Figure 8:**
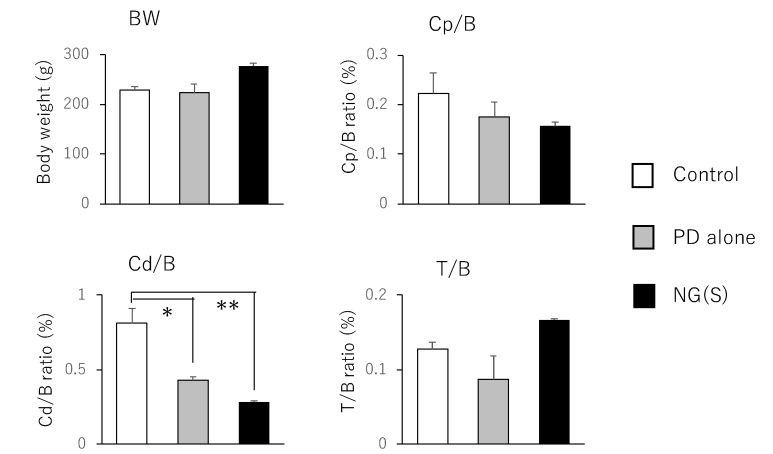
Parameter values associated with efficacy and toxic side effect at 5 mg PD eq./kg. BW, B: body weight, Cp: proximal colon weight, Cd: distal colon weight, T: thymus weight. The results are expressed as the mean ± S.E. (*n* = 3). * *p* < 0.05, ** *p* < 0.01.

**Figure 9:**
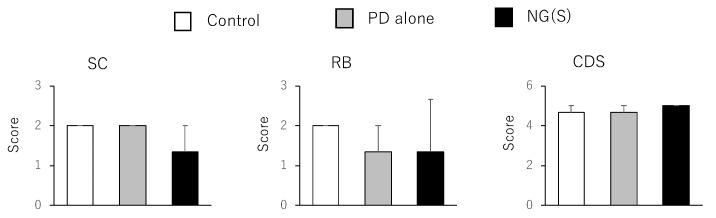
Scores associated with efficacy and toxic side effect at 5 mg PD eq./kg. SC: Stool consistency, RB: rectal bleeding, CDS: colonic damage score. The results are expressed as the mean ± S.E. (*n* = 3).

Therapeutic efficacy was evaluated using the ratio of (colonic weight)/(body weight) and colitis severity indices, which were reported previously. The results obtained at 5 mg PD eq./kg are shown in Figure 8; Figure 9, respectively. Among the control, PD alone and NG(S) groups, NG(S) had the smallest Cp/B and Cd/B values, indicating that NG(S) was more likely to reduce inflammation. The T/B ratio was the highest in the NG(S) group and not significantly different among the three groups. NG(S) may suppress the side effect of thymic atrophy. The increase in body weight may be due to the results based on the effectiveness. Regarding colitis severities at 5 mg PD eq./kg, the results obtained for SC, RB and CDS were not significantly different among the three groups.

**Figure 10:**
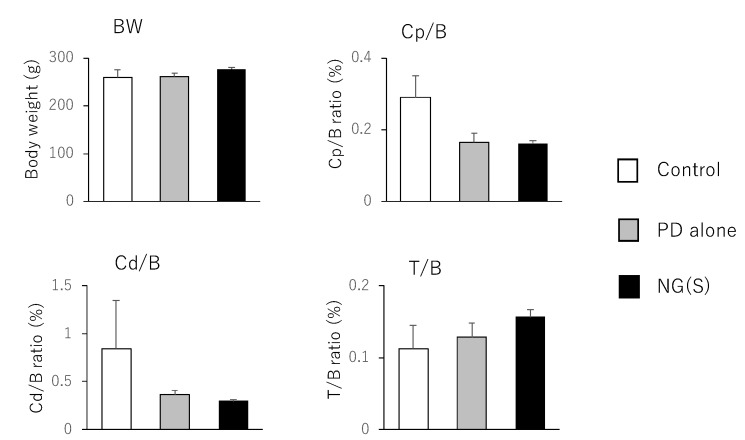
Parameter values associated with efficacy and toxic side effect at 10 mg PD eq./kg. BW, B: body weight, Cp: proximal colon weight, Cd: distal colon weight, T: thymus weight. The results are expressed as the mean ± S.E. (*n* = 4).

**Figure 11:**
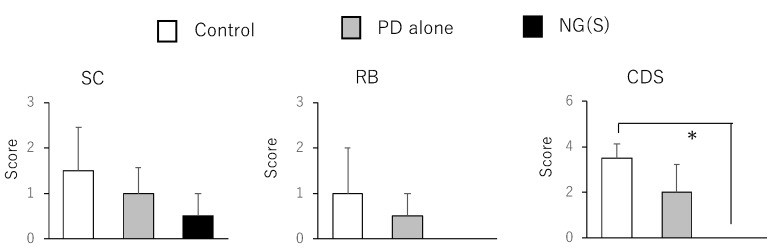
Scores associated with efficacy and toxic side effect at 10 mg PD eq./kg. SC: Stool consistency, RB: rectal bleeding, CDS: colonic damage score. The results are expressed as the mean ± S.E. (*n* = 4). * *p* < 0.05.

The results obtained for the treatment with 10 mg PD eq./kg are shown in Figure 10 and Figure 11. Body weight was the highest, while Cp/B and Cd/B values were the lowest in the NG(S) group; however, no significant differences were observed between the three groups. Furthermore, thymus weight was the highest in the NG(S) group, indicating that the nanogel suppressed the systemic toxic side effects of PD. Colitis severity was reduced to the greatest extent with NG(S). The colonic damage score was 0 with NG(S) and a significant difference was observed in CDS between the NG(S) and control groups. Although NG(S) did not clearly exhibit effectiveness at 5 mg PD eq./kg, marked anti-inflammatory efficacy occurred at 10 mg PD eq./kg. Moreover, thymic atrophy was suppressed slightly more with NG(S) than with PD alone, indicating that NG(S) was less toxic than PD alone. 

### 3.8. Histopathological Evaluation

**Figure 12:**
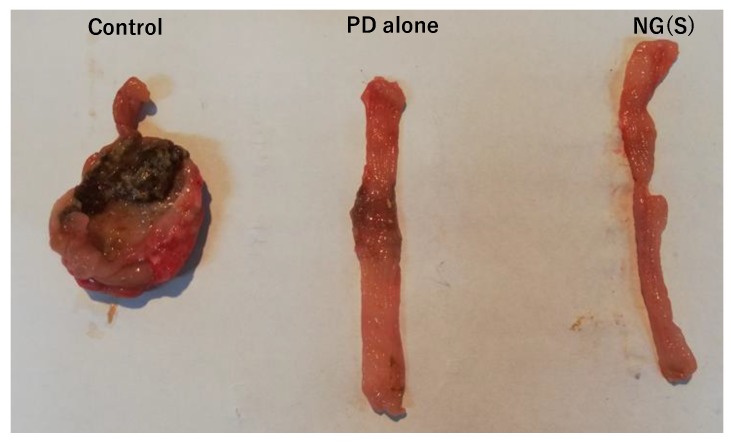
Representative images of distal colon on the assessment day at 10 mg PD eq./kg.

The region of the distal colon was removed from each group administered 10 mg PD eq./kg × 3 (Figure 12). The control group had large ulcers and colonic tissue was extensively deformed. A severe ulcer was noted at the restricted area in the PD alone group. In the NG(S) group, inflammation was negligible throughout colonic tissue. Therefore, NG(S) exerted the strongest anti-inflammatory effects.

**Figure 13:**
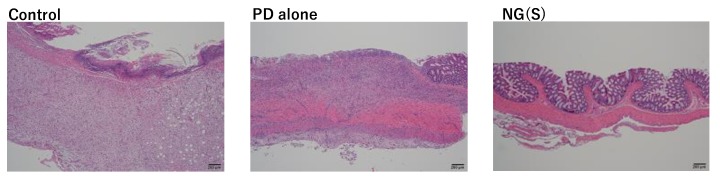
Representative H&E-stained distal colon sections at 10 mg PD eq./kg.

Cross-section images of the colonic site around the ulcer region were examined after H&E staining (Figure 13). Goblet cell depletion, necrosis and shedding of the epithelium, mild inflammatory cell infiltration, fibrotic granulation tissue formation and the loss of the muscle layer were observed at control thickened ulcer sites. Mucosal images were similar in the PD alone and control groups. The extent of the infiltration of inflammatory cells was also similar between the control and PD alone groups. However, the muscle layer persisted in the PD alone group. In the NG(S) group, a normal epithelial layer including goblet cells was clearly observed and there were no signs of inflammation. These results indicated that NG(S) suppressed inflammation in the colitis model.

Those in vivo studies revealed NG(S) could exhibit the enhanced efficacy and improved toxic side effects of PD. According to Xiao et al. [27], anionic nanoparticles and nanogels appear to adhere preferentially to inflamed colonic mucosa because positively charged proteins accumulate in such inflammatory regions. NG(S) was considered to work efficiently through such effects, which would be the future subject. 

## 4. Conclusions

In the present study, novel NG(S) composed of S-GCh-SP was prepared. The contents of the succinyl group and PD were 64% (mol/mol) per sugar unit and 13.7% (*w*/*w*), respectively, from ^1^H-NMR spectra. NG(S) had a submicron size of approximately 400 nm and a γ-potential of ca. −30 mV. NG(S) gradually released PD and the release rate was greater at the intestinal pH than at the gastric pH. In in vitro studies using the macrophage-like cell line Raw 264.7, NG(S) did not decrease cell viability, suggesting the safety of the nanogel. Cells exhibited the good uptake of NG(S), independent of LPS. In vivo efficacy tests were performed using rats with TNBS-induced colitis. NG(S) suppressed damage more than PD alone at 10 mg PD eq./kg; however, at 5 mg PD eq./kg, no significant differences were observed between the NG(S), PD alone and control groups. Furthermore, systemic toxicity was suppressed in the NG(S) group. The present results indicate the potential of NG(S) in the treatment of UC. 

## Figures and Tables

**Table 1:** Preparation condition and drug content of GCh-SP.

Preparation	GCh (mg)	SP (mg)	WSC (mg)	PD Content * (mg)
GCh-SP	100	100	500	23.0 + 2.2

* The result is expressed as the mean ± S.D. (*n* = 3).

**Table 2:** Preparation condition and drug content of S-GCh-SP.

Preparation	GCh-SP (mg)	SA (mg)	PD Content * (mg)
S-GCh-SP	150	1050	13.7 ± 1.4

* The result is expressed as the mean ± S.D. (*n* = 3).

**Table 3:** Particle size and ζ-potential of nanogels.

Preparation	Particle Size * (nm)	Zeta Potential * (mV)
NG(G)	408 ± 3	42.3 ± 0.4
NG(S)	396 ± 1	−27.9 ± 0.2

* The result is expressed as the mean ± S.D. (*n* = 3).
